# Are community-living and institutionalized dementia patients cared for differently? Evidence on service utilization and costs of care from German insurance claims data

**DOI:** 10.1186/1472-6963-13-2

**Published:** 2013-01-03

**Authors:** Larissa Schwarzkopf, Petra Menn, Reiner Leidl, Elmar Graessel, Rolf Holle

**Affiliations:** 1 Helmholtz Zentrum München, Institute of Health Economics and Health Care Management, Ingolstaedter Landstrasse, Neuherberg, 85764, Germany; 2 Clinic for Psychiatry and Psychotherapy, University Hospital Erlangen, Schwabachanlage 6, Erlangen, 91054, Germany

**Keywords:** Payer perspective, Social security system, Budget impact, Spending, Health care, Long-term care, Nursing care, ADL impairment, Care level, Care setting

## Abstract

**Background:**

Dementia patients are often cared for in institutional arrangements, which are associated with substantial spending on professional long-term care services. Nevertheless, there is little evidence on the exact cost differences between community-based and institutional dementia care, especially when it comes to the distinct health care services. Adopting the perspective of the German social security system, which combines Statutory Health Insurance and Compulsory Long-Term Care Insurance (payer perspective), our study aimed to compare community-living and institutionalized dementia patients regarding their health care service utilization profiles and to contrast the respective expenditures.

**Methods:**

We analysed 2006 claims data for 2,934 institutionalized and 5,484 community-living individuals stratified by so-called care levels, which reflect different needs for support in activities of daily living. Concordant general linear models adjusting for clinical and demographic differences were run for each stratum separately to estimate mean per capita utilization and expenditures in both settings. Subsequently, spending for the community-living and the institutionalized population as a whole was compared within an extended overall model.

**Results:**

Regarding both settings, health and long-term care expenditures rose the higher the care level. Thus, long-term care spending was always increased in nursing homes, but health care spending was comparable. However, the underlying service utilization profiles differed, with nursing home residents receiving more frequent visits from medical specialists but fewer in-hospital services and anti-dementia drug prescriptions. Altogether, institutional care required additional yearly per capita expenses of ca. €200 on health and ca. €11,200 on long-term care.

**Conclusion:**

Community-based dementia care is cost saving from the payer perspective due to substantially lower long-term care expenditures. Health care spending is comparable but community-living and institutionalized individuals present characteristic service utilization patterns. This apparently reflects the existence of setting-specific care strategies. However, the bare economic figures do not indicate whether these different concepts affect the quality of care provision and disregard patient preferences and caregiver-related aspects. Hence, additional research combining primary and secondary data seems to be required to foster both, sound allocation of scarce resources and the development of patient-centred dementia care in each setting.

## Background

Dementia is a common syndrome in advanced age and is characterized by a steady cognitive decline, resulting finally in a need for long-term care [1]. The incidence of dementia disorders increases exponentially beyond the age of 65, and the corresponding prevalence rates approximately double for every additional five years of age [2,3]. Around 7.2% of the German population aged 65 and older is thought to suffer from dementia – equivalent to ca. 1.2 million patients [2]. This figure is expected to double by 2050 as a result of demographic change alone [4].

According to the Federal Statistical Office, the German social security system, represented by the Statutory Health Insurance (hereafter SHI) together with the Compulsory Long-Term Care Insurance (hereafter LTCI), spent ca. €10.5 billion on treating dementia disorders in 2008. This equals ca. 8.4% of the entire public health and long-term care spending on individuals aged 65 and older [5]. Dementia is a substantial economic burden for the public health sector and, from this payer perspective, professional long-term care in an institutional setting is the crucial expense factor [6,7].

However, the exact difference in spending on long-term care services has seldom been quantified, and there is even less research that has analysed whether expenditures on health care services also differ between institutionalized and community-living dementia patients [8-11].

Within the German context, we know of only one study that has compared expenditures directly attributable to Alzheimer’s dementia in a sample of 123 institutionalized and 272 community-living individuals. Based on self-reports from a three-month recall period, yearly per capita expenditures were estimated at ca. €4,500 (community) versus €15,300 (nursing home) for SHI and LTCI [12]. These findings might not be generalizable as patients were mainly recruited via memory clinics and neurological practices. Moreover, comprehensive information on the volume of services used is lacking. Typically, utilization is easier to transfer to other countries and systems than cost figures [13].

To partly overcome some of these limitations, we assessed the entire service utilization and the corresponding expenditures for a large sample of community-living and institutionalized individuals suffering from any kind of dementia disorder via claims data from a prominent regional SHI fund. The living environment of the population, which might be nursing home or the community, is further referred to as the setting.

This paper aimed to

a) Compare yearly per capita utilization and costs for health and long-term care services between community-living and institutionalized dementia patients according to activities of daily living (hereafter ADL) dependency groups (care level)

b) Investigate systematic differences regarding the provision of distinct health care services within both care settings.

## Methods

### Data source and sample selection

AOK Bavaria is the leading SHI fund in the district of Middle Franconia covering about 50% of the population aged 65 years and older. It provided claims data from 2005 to 2007 for all insurants born before 1941 (i.e. at least 65 on 1 January 2006) and continuously insured until 2007 (i.e. survivors of 2005 and 2006). Data provision was carried out according to German data protection laws, and AOK Bavaria approved the use of the data set for the intended analyses. The selection process relied on 2005 and 2006 data. Service utilization and corresponding spending were compared for 2006.

Dementia patients were identified by means of physician diagnoses, hospital diagnoses and anti-dementia drug prescriptions by constructing ‘dementia quarters’. Each quarter with at least one documented dementia-specific ICD-10 code (‘F00’, ‘F01’, ‘F02’, ‘F03’ and ‘G30’) or with at least one filled prescription for a dementia-specific drug (ATC codes N06DA (cholinesterase inhibitors) and N06DX01 (memantine)) was defined as a dementia quarter. To avoid false-positive classification as a dementia patient, we only considered individuals with a minimum of three dementia quarters out of four consecutive quarters [14,15]. At least one of these dementia quarters was required to be documented in 2006. Using this approach, we identified 9,147 dementia patients. A detailed description of the selection process is described elsewhere [16].

This sample was classified as community-living or institutionalized in a nursing home based on a distinct component of long-term care insurance called ‘institutional care’. We decided that the starting date of payments for ‘institutional care’ would be synonymous with transfer to a nursing home. Moreover, we assumed institutional care only to be affordable with LTCI support [12]. Thus, all individuals without LTCI support were allocated to the community setting. In one special case, an institutionalized individual with LTCI support in 2005 received no extension of the long-term care entitlement in 2006. We dropped this insurant. In this way, we identified 5,584 insurants living in the community setting and 2,934 insurants living in an institutional setting throughout 2006. In addition, 682 individuals were found to have transferred to a nursing home during the course of 2006, and 46 received LTCI support for ‘institutional care in homes for the handicapped’.

Our interest was a cross-sectional comparison of the community and the institutional setting. Therefore, we excluded incidental nursing home residents in 2006 because they represented an independent third group that could not be allocated definitively to one of the settings of interest. We also disregarded individuals residing in homes for the handicapped because their care needs most probably differed from those of individuals residing in regular nursing homes. Hence, our final sample consisted of 8,418 dementia patients.

Within the German long-term care system, the need for care is reflected by three care levels, which account for the daily support required in ADL. Care level 1 reflects mild dependency, care level 2 moderate dependency and care level 3 severe dependency.

Based on previous studies, we expected a different degree of ADL dependency for individuals in each setting [12,17,18] and regarded an unequal care level distribution as a potential source of bias. For a comprehensive comparison, we therefore further stratified both populations according to care level, using the care level assigned on 30 June as the classification criterion.

### Assessment of service utilization and expenditures

#### The German health and long-term care system

In Germany, health and long-term care insurance are a legal obligation, and about 85% of the resident population is insured within statutory funds. These are characterized by pay-as-you-go financing and income-dependent but risk-independent contributions [19].

There are no different funding sources for community-living and nursing home-based individuals, but the responsibility of SHI versus LTCI is defined by the type of service required: SHI covers acute health care needs and temporary nursing needs (e.g. household support after a fracture). It is designed as full coverage insurance providing almost free access to a broad range of medical services [19]. In contrast, LTCI accounts for durable ADL impairment and has only a supportive character. Hence, long-term care services are only reimbursed up to a fixed ceiling amount, which depends, first, on the type of service utilized and, second, on the patient’s ‘care level’ (ADL dependency group) [20].

LTCI data and SHI data are managed under one umbrella, but the corresponding services are financed by either LTCI contributions or SHI contributions.

#### SHI services

With regard to SHI, we assessed service utilization and corresponding expenditures for hospital care, outpatient treatment by physicians, medication, non-physician services, medical aids, home health care and rehabilitation. The per capita expenditures for 2006 therefore equal the sum of expenditures within the distinct service categories.

Whenever a treatment only partly took place in 2006, we calculated time of treatment within the year of observation. Then, expenditures were attributed proportionally to time of resource use.

Utilization of physician services is reported on the level of visits. Each visit is connected with multiple services billed via a fee for service system which assigns a specific score to each service. The corresponding counter value of services is calculated by multiplying this score by a point value variable by quarter and the physician’s area of specialization.

Medication use refers to the number of prescribed drugs. The respective pharmacy retail prices are available from the Scientific Institute of the AOK. As well as medication use and expenditures in general, we assessed prescriptions and spending for dementia-specific drugs in particular.

Hospitals provide inpatient treatment, emergency care and certain outpatient services, all accounted for within the category of hospital treatment. Service utilization is described as the sum of days with any kind of hospital treatment. Spending on hospital services is documented directly in the claims data.

Expenditures on non-physician services and home health care refer to prescriptions. A prescription for non-physician services covers a fixed number of treatment sessions, whereas home health care is limited to a distinct time horizon. The absolute payment amount per prescription depends, first, on the number of contacts between patient and provider and, second, on the type of services provided. These details are not traceable; instead, only the total sum accrued for the delivered services is reported. Therefore, the number of prescriptions gives only a vague idea of effective resource utilization.

#### LTCI services

For nursing home residents, the LTCI offers ‘permanent institutional care’, which includes basic and medical nursing and social care but does not account for board and lodging. Community-living individuals can choose between ‘professional ambulatory nursing care’, ‘allowance for nursing care’, which represents a transfer payment to informal caregivers, or a combination of both services. Additionally, they receive short-term institutional nursing care, day or night care and replacement caregiving, if required.

However, not every insurant automatically has an entitlement to LTCI support. In order to benefit from LTCI payments, individuals with ADL impairment have to file an application to their SHI. Then, their need for care is appraised by the Medical Review Board – an independent statutory corporation performing advisory functions to SHI and LTCI that is directly supervised by the state’s ministry of social affairs - according to a standardized procedure. This assessment basically accounts for ADL needs but does not explicitly address dependency due to cognitive impairment [21].

The Code of Social Law defines the following care levels. Care level 1 (mild dependency) requires a minimum of 90 minutes support per day, of which at least 45 minutes is dedicated to basic care. Care level 2 (moderate dependency) requires a minimum of 180 minutes support per day, of which at least 120 minutes is dedicated to basic care. Care level 3 (severe dependency) requires a minimum of 300 minutes support per day, of which at least 240 minutes is dedicated to basic care.

At the end of the process, either the applicant is assigned to one of these care levels or the application is rejected. In the latter case, all long-term care services have to be financed out-of-pocket. Otherwise, the applicants receive a set amount of monthly support, which increases the higher the care level.

As these tariffs are fixed, claims data only provide information on the utilization of a certain LTCI service per se, but not on the intensity of service utilization.

#### Statistical analysis

To compare care level-specific utilization patterns and insurance expenditures of institutionalized and community-living dementia patients, we applied a separate generalized linear model (hereafter GLM) for each care level. As individuals without care level assignment were only observed in the community setting, we constructed particular models for this subpopulation only. In all these models, the influence of setting was investigated adjusting for age, gender, the interactions ‘age*gender’, ‘age*setting’ and ‘gender*setting’ as well as for proximity to death and comorbidity.

Proximity to death was reflected by a dummy variable which reported whether an individual survived the year 2007 or not. Comorbidity was assessed by the Charlson Comorbidity Index [22]. To indicate the comorbidity burden at the beginning of 2006, we accounted for all inpatient and outpatient diagnoses documented in the second half of 2005.

The inclusion in the models of interactions with setting complicated a straightforward interpretation of the setting effect. Therefore, we also performed simple GLMs without interaction terms. Here, the p-value of setting can be interpreted directly. These estimates differed only marginally from those of the full models and, given this high accordance, we considered a significant difference in the simple model to be transferable to the extended model.

Age, gender and comorbidity burden differed to some extent between the different strata. To enable a comparison across the different strata, we adjusted the strata-specific figures on the average age, gender and comorbidity profile across the entire study population. This implied a mean age of 81.6 years, a female quota of 74.6% and a mean Charlson Index of 4.0 for all analyses.

We analysed the average volume of service use at the per capita level for each category assuming a negative binomial distribution with log link.

Expenditures were estimated based on two different approaches depending on the service category concerned. Approach 1, a one-step gamma model with log link, was applied for services used by almost every patient. These were general practitioner, medication, SHI expenditures, LTCI expenditures and expenditures for the social security system. To avoid excluding the few patients without expenditures from the analyses, we assigned them a small positive amount (€10 per person).

For the categories medical specialist, hospital treatment, non-physician services, medical aids, home health care, rehabilitation and the distinct long-term care services, we followed approach 2. Here, we calculated two-part regression models [23], which first assess the probability of positive expenditures via logistic regression (part 1) and subsequently estimate the amount expended for the individuals with positive spending by a gamma model with log link (part 2). To derive per capita expenditures, we multiplied the estimated probabilities for positive expenditures from part 1 by the predicted expenditures per user from part 2. Group differences combined for both parts of the model were evaluated using recycled predictions based on setting as the coefficient of interest [24].

Owing to the stepwise calculation, approach 2 provides two p-values, the first referring to differences in the probability of service use (part 1), and the second referring to expenditure differences among service users (part 2). We defined the effect of setting as statistically significant if both estimates pointed in the same direction and if at least one of these p-values was significant.

Another effect of the stepwise approach was a slight difference between SHI expenditures and expenditures for the social security system derived from the one-part model and the sum of mean expenditures in the associated expenditure categories.

Supplementary to the care level-stratified analyses, we constructed an overall GLM that considers the whole study population. In addition to the covariates described previously, this overall model accounted for the covariates care level, ‘care level*gender’, ‘care level*age’ and ‘care level*setting’. LTCI expenditures were also estimated within a two-part model. Apart from these modifications, the analyses followed the approaches described above.

A significance level of 5% was used for all analyses, which were performed with the software package SAS, version 9.2.

## Results

### Patient characteristics

Table 1 describes the patient population according to care level assignment on 30 June, which reflects ADL impairment. We found a higher level of physical independence in the community setting. Some 3,325 community-living individuals but only one institutionalized individual were not assigned to any care level. Also, the quota of patients assigned to care levels 2 or 3 was substantially higher in nursing homes. Across all care levels, nursing home residents were around two years older than community-living individuals and the share of females was increased.

**Table 1:** Baseline characteristics

	**Institution**	**Community**	**Entire sample**
***Entire population***			
N	2,934	5,484	8,418
Age	84.3 (7.3)	80.1 (7.1)	81.6 (7.5)
Charlson Index	3.9 (2.2)	4.1 (2.4)	4.0 (2.3)
Females (as a %)	2,453 (83.6)	3,827 (69.9)	6,280 (74.6)
Death in 2007 (as a %)	747 (25.5)	719 (13.1)	1,466 (17.4)
***No care level***			
N	1*	3,325	3,326
Age		78.8 (6.6)	78.8 (6.65)
Charlson Index		4.0 (2.4)	4.0 (2.4)
Females (as a %)		2,303 (69.3)	2,304 (69.3)
Death in 2007 (as a %)		257 (7.7)	257 (7.7)
***Care level 1***			
N	705	1,059	1,764
Age	83.4 (7.2)	81.8 (7.0)	82.4 (7.1)
Charlson Index	3.9 (2.2)	4.1 (2.4)	4.0 (2.3)
Females (as a %)	561 (79.6)	762 (72.0)	1,323 (75.0)
Death in 2007 (as a %)	141 (20.0)	153 (14.4)	294 (16.7)
***Care level 2***			
N	1,201	729	1,930
Age	84.1 (7.4)	82.3 (7.3)	83.5 (7.1)
Charlson Index	3.9 (2.3)	4.4 (2.4)	3.8 (2.2)
Females (as a %)	991 (82.5)	502 (68.9)	1,493 (77.4)
Death in 2007 (as a %)	293 (24.4)	180 (24.7)	473 (24.5)
***Care level 3***			
N	1,028	371	1,399
Age	85.3 (7.3)	83.0 (7.7)	84.7 (7.5)
Charlson Index	3.7 (2.2)	4.1 (2.4)	3.7 (2.3)
Females (as a %)	900 (87.6)	260 (70.1)	1,160 (82.9)
Death in 2007 (as a %)	313 (30.5)	129 (34.8)	442 (31.6)

Data are mean (standard deviation) unless noted otherwise.

* Individual excluded from further analyses.

The comorbidity burden according to the Charlson Index was slightly higher in the community but, in both settings, the index score altered little between the care level strata. Irrespective of care setting, the probability of dying during the year following the observation increased with rising ADL impairment.

### Care level-based comparison of service utilization

The user quotas in Table 2 show that almost every dementia patient visited general practitioners and took medication. Other SHI services commonly used were treatment by medical specialists and prescriptions for medical aids. In contrast, non-physician services and especially rehabilitation were only relevant for a minority.

**Table 2:** User quota of health care services in 2006 according to care levels

**Setting-specific utilization quota of health care services according to care level adjusted for age, gender, comorbidity, mortality and single interactions**										
	**No care level**	**Care level 1**			**Care level 2**			**Care level 3**		
	**Community**	**Institution**	**Community**	**p-value**	**Institution**	**Community**	**p-value**	**Institution**	**Community**	**p-value**
	**(n=3,325)**	**(n=705)**	**(n=1,059)**		**(n=1,201)**	**(n=729)**		**(n=1,028)**	**(n=371)**	
General practitioner (v)	99.1%	100.0%	99.5%	ns	100.0%	100.0%	ns	100.0%	99.5%	ns
Medical specialist (v)	85.5%	86.6%	79.3%	**	87.1%	72.9%	***	77.8%	61.6%	***
Medication (p)	99.1%	100.0%	99.9%	ns	100.0%	100.0%	ns	99.7%	100.0%	ns
*of which anti-dementia drugs (p)*	*16.5%*	*10.2%*	*21.2%*	***	*9.5%*	*15.4%*	***	*5.0%*	*8.5%*	*
Hospital treatment (d)	35.1%	40.0%	43.2%	ns	46.3%	45.5%	ns	39.7%	41.3%	ns
Non-physician services (p)	17.8%	25.9%	22.1%	*	38.3%	29.7%	**	31.6%	33.3%	ns
Medical aids (p)	42.0%	84.8%	54.0%	***	94.6%	59.8%	***	96.9%	70.9%	***
Rehabilitation	5.4%	1.4%	7.2%	***	0.9%	3.6%	**	***estimation not possible***		./.
Home health care	16.1%	*not provided*	35.1%	./.	*not provided*	40.9%	./.	*not provided*	44.8%	./.

v=visits; p=prescriptions; d=days.

* p≤0.05; ** p ≤0.001, *** p≤0.0001; ns=not significant.

Comparing the percentage of community-living and institutionalized users for the three distinct care level strata reveals that the quota of nursing home-based users was higher regarding medical specialists and medical aids and, except for care level 3, regarding non-physician services, too. The percentage of community-living service users was higher for each care level regarding rehabilitation and dementia-specific drugs.

Table 3 focuses on the intensity of per capita service utilization and shows that nursing home residents assigned to care levels 1 or 2 used general practitioners, medical specialists, non-physician services, medical aids and drugs more frequently than their community-living counterparts, who on the other hand required anti-dementia drugs, hospital treatment and rehabilitation on a larger scale. In care level 3, the utilization of hospital treatment, anti-dementia drugs and non-physician services was comparable in both settings. Similar to care levels 1 and 2, physician visits and medical aid prescriptions were higher in nursing homes but, in contrast to the lower care levels, drug intake was increased in the community.

**Table 3:** Per capita health care service utilization in 2006 according to care levels

**Setting-specific utilization volume of health care services per capita according to care level adjusted for age, gender, comorbidity, mortality and single interactions**										
	**No care level**			**Care level 1**			**Care level 2**			**Care level 3**		
	**Community**	**Institution**	**Community**	**p-value**	**Institution**	**Community**	**p-value**	**Institution**	**Community**	**p-value**	
	**(n=3,325)**	**(n=705)**	**(n=1,059)**		**(n=1,201)**	**(n=729)**		**(n=1,028)**	**(n=371)**		
General practitioner (v)	28.9	52.4	33.2	***	55.0	35.8	***	54.5	40.8	***
Medical specialist (v)	10.8	12.6	8.7	***	15.0	7.2	***	12.3	6.4	***
Medication (p)	25.1	36.5	32.5	***	42.1	37.9	**	38.4	46.8	***
of which anti-dementia drugs (p)	0.7	0.5	1.0	**	0.4	0.8	*	0.2	0.5	ns
Hospital treatment (d)	5.8	6.3	8.4	*	7.6	8.3	ns	6.4	7.0	ns
Non-physician services (p)	0.5	1.2	0.9	*	2.0	1.5	*	1.8	2.0	ns
Medical aids (p)	0.8	3.3	1.2	***	5.0	1.6	***	6.3	3.6	***
Rehabilitation (d)	1.3	0.1	1.8	***	0.3	4.2	**	0.0	0.2	ns
Home health care (p)	1.3	*not provided*	3.3		*not provided*	3.7		*not provided*	4.3	./.

v=visits; p=prescriptions; d=days.

* p≤0.05; ** p ≤0.001, *** p≤0.0001; ns=not significant.

### Development of utilization profiles across care levels

As outlined in Table 3, the per capita demand of community-living dementia patients for general practitioners, drugs, non-physician services and medical aids increased steadily with rising ADL dependency but, in parallel, the use of medical specialists decreased. The number hospital days and dementia-specific drug prescriptions also declined continuously from care level 1 to care level 3, but here individuals with no care level entitlement had lower volumes of service use than those assigned to a care level.

In the institutional setting, we observed a linear increase in prescriptions for medical aids from care level 1 to care level 3 and a linear decrease in prescriptions for dementia-specific drugs. Within all other SHI services, dementia patients in care level 2 presented a higher volume of per capita utilization than those in care levels 1 and 3.

### Service expenditures

Compared with the community setting LTCI spending was remarkably increased in nursing homes, as shown in Table 4. The additional expenses amounted to ca. €8,900 in care level 1, ca. €7,700 in care level 2 and ca. €5,800 in care level 3. In contrast, SHI spending was higher in the community by ca. €1,100, ca. €200 and ca. €1,100 respectively.

**Table 4:** Per capita expenditures on health and long-term care according to care level

**Setting-specific expenditures (€) on health care and long-term care services per capita according to care level adjusted for age, gender, comorbidity, mortality and single interactions**										
	**No care level**	**Care level 1**			**Care level 2**			**Care level 3**		
	**Community**	**Institution**	**Community**	**p-value**	**Institution**	**Community**	**p-value**	**Institution**	**Community**	**p-value**
	**(n=3,325)**	**(n=705)**	**(n=1,059)**		**(n=1,201)**	**(n=729)**		**(n=1,028)**	**(n=371)**	
Health insurance*	**4,018**	**4,666**	**5,830**	**s**	**6,162**	**6,395**	**ns**	**5,962**	**7,139**	**s**
without home health care*	***3,593***	***4,681***	***4,888***	***ns***	***6,159***	***5,425***	***s***	***5,965***	***6,449***	***ns***
General practitioner	515	777	634	s	771	701	s	746	805	s
Medical specialist	380	366	350	s1	387	237	s	282	194	s1
Medication	824	1,221	1,195	s	1,638	1,416	s	1,762	2,251	s
*of which anti-dementia drugs*	*125*	*81*	*177*	*s1*	*15*	*118*	*s2*	*29*	*90*	*s*
Hospital treatment	1,573	1,751	2,199	s2	2,333	2,425	ns	1,908	2,037	ns
Non-physician services	50	182	127	s	329	281	s2	301	452	s2
Medical aids	85	391	150	s	676	201	s	932	632	s
Rehabilitation	180	28	246	s2	29	128	s2	**estimation not possible**		./.
Home health care	390	*not provided*	899	./.	*not provided*	1,000	./.	*not provided*	723	./.
**Long-term care insurance**	**0**	**12,068**	**3,173**	**s**	**14,850**	**7,168**	**s**	**16,714**	**10,929**	**s**
*of which transfers to informal care*	*not provided*	*not provided*	*1,752*	*./.*	*not provided*	*3,370*	*./.*	*not provided*	*5,561*	*./.*
Social security system*	***4,018***	***16,982***	***9,047***	***s***	***21,208***	***13,795***	***s***	***22,921***	***18,437***	***s***

s=significant in parts 1 and 2 of the two-part model or in the one-part model; s1=significant in part 1 of the two-part model; s2=significant in part 2 of the two-part model; ns=not significant.

* Results of model estimation; Addition of mean costs per category yields slightly different figures.

These overall SHI expenses also account for ‘home health care’, which is by definition not offered to institutionalized individuals. If only services available to both patient populations were looked at, the difference in expenditures basically evened out.

A detailed look at the distinct SHI services revealed that expenditures on dementia-specific medication, hospital treatment and rehabilitation were higher within all care levels in community-living individuals and expenditures on general practitioners and medical specialists were higher in institutionalized individuals. Regarding non-physician services, medical aids and overall medication, the picture is less clear, because nursing home residents incurred higher spending in care levels 1 and 2 but lower spending in care level 3.

From care level 1 to care level 3, the LTCI expenditures rose by ca. €7,800 in the community and by ca. €4,600 in nursing homes. The corresponding additional amount for SHI was ca. €1,300 in both settings. As a consequence, the excess spending for institutional care concurrently declined the higher the care level, albeit the overall spending for both populations increased.

### Comparison across all care levels

According to Table 5, only the categories medication and hospital treatment required comparable financial resources, if the entire population of community-living dementia patients is compared with their institutionalized counterparts. Expenditures on general practitioners, medical specialists, non-physician services and medical aids were higher in nursing homes but expenditures on anti-dementia drugs and rehabilitation were higher in the community. Altogether, the yearly per capita spending of SHI on institutionalized dementia patients was increased by ca. €200 and the yearly per capita spending of LTCI by ca. €11,200.

**Table 5:** Per capita service utilization and expenditures (€) in 2006 across all care levels

**Service utilization and expenditures (€) per dementia patient across all care levels adjusted for age, gender, comorbidity, mortality and care level**										
	**Institution (n=2,934)**		**Community (all) (n=5,584)**		**Difference**		**Community (care level) (n=2,159)**		**Difference**	
	**Volume**	**Costs**	**Volume**	**Costs**	**Costs**	**p-value**	**Volume**	**Costs**	**Costs**	**p-value**
Health insurance*		**5,693**		**5,466**	**227**	**s**		**6,424**	**−731**	**s**
without home health care*		***5,678***		***4,759***	***919***	***ns***		***5,533***	***145***	***ns***
General practitioner (v)	54.3	761	33.2	626	135	s	37.6	705	56	s
Medical specialist (v)	13.0	327	8.8	310	17	s	7.2	264	63	s
Medication (p)	38.8	1,636	33.0	1,270	366	ns	41.1	1,569	67	ns
*of which anti-dementia drugs (p)*	0.3	*49*	0.7	*129*	*−80*	*s*	0.6	*131*	*−82*	*s*
Hospital treatment (d)	6.2	1,904	7.2	1,990	−86	ns	7.5	2,253	−349	s1
Non-physician services (p)	1.7	295	1.1	189	106	s2	1.7	281	14	s2
Medical aids (p)	5.3	742	1.5	216	526	s	2.6	303	439	s
Rehabilitation (d)	0.1	16	1.1	140	−124	s2	0.7	128	−112	s2
Home health care (p)	not provided		2.8	694	−694	./.	4.1	893	−893	./.
**Long-term care insurance**		**15,340**		**4,159**	**11,181**	**s**		**6,810**	**8,530**	**s**
*of which transfers to informal care*				*2,082*	*−2,082*	*./.*		*3,414*		*./.*
Social security system*		***21,388***		***9,765***	***11,623***	***s***		***13,477***	***7,911***	***s***

v=visits; p=prescriptions; d=days.

s=significant in parts 1 and 2 of the two-part model or in the one-part model; s1=significant in part 1 of the two-part model; s2=significant in part 2 of the two-part model; ns=not significant.

* Results of model estimation; Addition of mean costs per category yields slightly different figures.

Additionally, we performed this comparison for the subpopulation of dementia patients with care level assignment. Now, the institutionalized population had reduced SHI spending by ca. €700 and increased LTCI spending by ca. €8,500.

## Discussion

Within the German social security system, the yearly per capita health and long-term care expenses on institutionalized dementia patients amount to ca. €21,400, of which almost three quarters is attributable to professional long-term care. The overall spending on community-living dementia patients is ca. €9,800 – less than half as much – and at ca. €4,200, the corresponding long-term care expenses are substantially lower.

A comprehensive comparison of both settings according to ADL dependency reveals a trend of rising costs of care with greater physical impairment. Comparing mildly dependent patients with severely dependent ones, the increase – including, again, SHI and LTCI – is ca. €9,400 (+104%) in the community and ca. €5,900 (+35%) in nursing homes. In both patient groups, the long-term care expenses rise more than the health care expenses: SHI spending on nursing home residents increases by ca. €1,300 (+28%) but LTCI spending by ca. €4,600 (+ca.38%). The corresponding figures for community-living individuals are ca. €1,300 (+22%) in SHI and ca. €7,800 (+245%) in LTCI.

A rise in expenditures from care level to care level was by and large expected, even though it has to be considered as artificial regarding long-term care: LTCI tariffs are fixed by legal definition and, hence, different from SHI payments, partially detached from effective needs.

Focusing on the distinct SHI categories, the utilization of routine services increases at higher care levels, but more complex services (medical specialist, hospital, rehabilitation) are utilized less frequently. This might indicate that specialized, interactive – and therefore costly – treatment approaches that aim to restore the patients’ capabilities for independent living are perhaps not considered promising any longer when a certain degree of ADL dependency is reached.

Comparing the health care expenditures between both settings, it has to be kept in mind that SHI services include ‘home health care’, which accounts for short-term medical nursing and temporary household support in community-living individuals. These services are by definition not available for nursing home residents. To fairly judge the costs of health care service provision within the distinct settings, we thus contrasted SHI spending exclusive of expenditures on home health care. In doing so, we found no significant difference between both patient populations.

Despite the fact that overall health care expenses for community-living and institutionalized dementia patients are comparable, the underlying utilization patterns show some notable differences.

First, community-living dementia patients have more inpatient days than their institutionalized counterparts. Our result is similar to the findings of two US-based studies which reported more frequent hospital admissions in community-living elderly patients [9,25]. This suggests that some acute illnesses overburden informal family caregivers but can be brought under control by professional nursing staff in institutions, which renders hospitalization dispensable sometimes. A further contribution to extended inpatient days in community-living dementia patients might be the relieving effect on informal family caregivers.

Obviously, community-living dementia patients face an increased risk of inpatient treatment, and SHI should consider offering caregiver support programmes and case management approaches to prevent avoidable hospitalizations.

Second, contrary to previous studies [8,12], there is no evidence for worse access of nursing home residents to medical specialists. The contact frequency is comparable with most medical specialists and even significantly increased regarding internal specialists and neurologists. Nursing home residents might have more regular access to specialized medical care because some medical specialists visit their institutions according to a fixed schedule. Thus, it seems to be easier to get an appointment for all patients in need. As a consequence, institutionalized dementia patients might receive more systematic treatment of physical comorbidity and dementia-related symptoms.

To achieve a corresponding advantage for the community-living population, it would be advisable to develop recommendations on which medical specialists have to be visited regularly. Moreover, especially in rural areas, innovative remuneration schemes might be required to secure a sufficiently dense network of specialized care.

Third, a higher quota of community-living than of institutionalized dementia patients receives dementia-specific pharmacological treatment. A corresponding difference was also observed by Reese et al. [12]. Perhaps physicians feel more obliged to tap the full potential of pharmacological treatment in community-living individuals, whose families most likely feel overburdened by worsening dementia symptoms, than in institutionalized dementia patients, whose disease progression is witnessed by professional nursing staff with a larger personal distance.

Although dementia-specific drugs can achieve temporary symptomatic improvement and postpone the inevitable disease progression, only 12.8% of our study sample received cholinesterase inhibitors or memantine. The minor relevance of anti-dementia drug therapy was also stated within another recent German claims data analysis [26].

To improve adequate pharmacological treatment, physicians need to be more aware of the beneficial aspects of anti-dementia drugs. To this end, they could be offered targeted vocational training.

To better judge the informative value of our findings, the strengths and weaknesses of claims data-based studies have to be considered.

First, claims data lack information on cognitive status [6]. Thus, we had no means of stratifying our analyses according to dementia severity itself. Given sufficient evidence that dementia progression increases the risk of institutionalization, it must be concluded that institutionalized patients are at a more advanced disease stage. If accepting a relation between cognitive and physical decline [27], the care level strata can serve as a rough proxy for disease severity and at least partially reflect how worsening symptoms of dementia affect health and long-term care expenditures in both settings.

Second, the documented diagnoses cannot be verified via medical examinations; thus, their accuracy is not absolutely guaranteed [28-31]. To avoid including false-positive cases, we required multiple dementia quarters. To incur multiple dementia quarters, an individual needs regular contact with the health care system. This implies the selection of dementia patients, who in principle have a higher morbidity burden than the entire population of insurants with dementia. Hence, our estimates most probably represent a ceiling amount for per capita expenses for dementia patients within the German social security system.

Third, claims data barely provide information on educational level, economic situation and family status, but these factors influence the intensity of demand for professional long-term care. We assume that the denser the informal care network, the lower the need for professional support. The LTCI component ‘allowance for nursing care’ allows the identification of whether informal care is available, but does not assess the informal care hours exactly. Among the community-living individuals in our sample, ‘allowance for nursing care’ accounts for approximately 50% of LTCI spending, and almost every insurant receives corresponding payments. Obviously, informal care plays a crucial role for community-living dementia patients and professional care has a barely supplementary character. In contrast, care provision is almost totally professional in nursing homes. The dimensions of confounding resulting from a different degree of professionalization remain unclear.

On the other hand, claims data-based patient samples seem to be better suited to describing routine care than study samples recruited from neurological wards and memory clinics [10,12] because the patient clientele is less selective. Moreover, claims data provide comparatively reliable figures because, contrary to survey-based estimates, recall bias, cost unit definition and extrapolation rules are dispensable. Third, claims data-based estimates are less influenced by single outliers because of their large sample sizes.

Despite its limitations, to our knowledge, our analysis is one of the most comprehensive comparisons between community-living and institutionalized dementia patients from a payer perspective yet undertaken. It addresses the different case mix in nursing homes and the community by care level-stratified analyses adjusting for age, gender, morbidity and mortality to ensure the best possible comparability between settings. Moreover, it presents costs of care on a disaggregated level and itemizes the distinct health care services. This allows easy tracing of which components first and foremost trigger SHI spending. As comprehensive care strategies have to consider the health and long-term care needs of dementia patients, the chosen perspective includes both the SHI and the LTCI, although they are basically independent insurance branches. In doing so, our study revealed that community-based care for dementia patients is the cost-saving option for the German social security system because of significantly lower long-term care expenditures.

However, it has to be clearly stressed that the presented payer-based view is not necessarily in accordance with the societal perspective. Here, production losses and expenditures on behalf of the patients and their families would also be accounted for. For example, food and lodging in nursing homes have to be financed privately. In 2007, nursing home fees averaged €1,900 per month for care level 1, €2,300 for care level 2 and €2,800 for care level 3 [20]. Thus, only around 50% of monthly expenditures are covered by LTCI tariffs. This comes close to the results of a Belgian study, in which around 55% of expenditures for institutionalized dementia patients were borne by their families [8]. A fair comparison should thus consider food and lodging in community-living individuals. This approach was followed by Kuo et al. and yielded 2.5 times higher spending in the community [10].

Moreover, community-based care is associated with substantial costs of informal care [10,12,32], which most probably overcompensate for savings on professional long-term care services. Altogether, the economic advantage of community-based dementia care cannot be judged unambiguously from a societal perspective.

In addition, pure financial figures abstract away from quality of service delivery, caregiver-related issues and patient preferences. Future research combining primary and secondary data could account for these manifold aspects of dementia care. This would allow a more comprehensive judgement on the preferability of both settings and help to understand why – despite shared clinical and demographic characteristics – some dementia patients are cared for in the community and others in nursing homes.

## Conclusion

Community-living and institutionalized dementia patients differ regarding their demographic and clinical characteristics, and both settings are obviously not equivalent options for any particular patient. Thus, efforts aiming to postpone nursing home placement have to be scrutinized critically even though community-based care is the cost-saving option from a payer perspective. To improve a sound allocation of scarce resources, it seems to be more advisable to develop comprehensive care concepts for both settings. In this regard, it has to be analysed whether the indicated existence of setting-specific health care strategies – represented by different service utilization profiles of community-living and institutionalized individuals – affects the quality of care provision. Ideally, patient preferences and caregiver-related issues should also be integrated into a comprehensive picture on patient-centred dementia care. This ambitious goal requires future study approaches combining primary data on qualitative aspects and secondary data on costs.

## Competing interests

For LS, PM, RL, RH (Helmholtz Zentrum München) and EG (University Hospital Erlangen - Clinic for Psychiatry and Psychotherapy), their institutions received support from the funding organizations for the submitted work. RL and EG received an honorarium from one of the funding organizations for presenting data or participating in an advisory board.

## Authors’ contributions

LS analysed the data and drafted the manuscript. PM decided on the definite statistical approach and finalized the statistics section of the manuscript. Together with RH, both determined the study design and research questions to be answered. RL and EG advised on health care system-related issues and confirmed the constitution of the final study design. All authors contributed to the manuscript and approved the final version of the manuscript. LS is guarantor.

## Pre-publication history

The pre-publication history for this paper can be accessed here:

http://www.biomedcentral.com/1472-6963/13/2/prepub
